# Technology-Based Prehabilitation for Patients With Cancer Before Elective Treatment: Protocol for a Scoping Review

**DOI:** 10.2196/86610

**Published:** 2026-05-12

**Authors:** Grace Woods, Christopher J Gaffney, Ondrej Ryska, Lawrence D Hayes

**Affiliations:** 1 Faculty of Health & Medicine, Lancaster University Medical School, Lancaster University, Sir John Fisher Drive, Lancaster, LA1 4AT, United Kingdom, 01524 65201; 2 Lancashire Teaching Hospitals NHS Foundation Trust, Rosemere Cancer Centre, Preston, United Kingdom; 3 University Hospitals of Morecambe Bay NHS Foundation Trust, Kendal, United Kingdom

**Keywords:** prehabilitation, cancer, technology, telemedicine, mobile health, digital health

## Abstract

**Background:**

Advances in cancer treatment have improved survival rates; however, patients continue to experience significant treatment-related side effects, leading to reduced quality of life. Prehabilitation is an intervention that occurs before treatment and can improve patients’ functional capacity, recovery, and well-being through exercise, nutrition, and psychological support. Typical hospital-based prehabilitation is not accessible to all patients due to geographical, socioeconomic, and time-related barriers. Technology-based approaches, including eHealth and mobile health (mHealth) interventions, may overcome these barriers by enabling remote, patient-centered delivery. However, the current evidence base is heterogeneous and lacks synthesis regarding feasibility, acceptability, and outcomes.

**Objective:**

This protocol for a scoping review aims to outline how we will systematically map and synthesize the evidence on technology-based prehabilitation interventions for people with cancer to identify intervention designs, assess feasibility and accessibility, and highlight knowledge gaps to guide future research and practice.

**Methods:**

The review will follow the Joanna Briggs Institute (JBI) methodology and PRISMA-ScR (Preferred Reporting Items for Systematic Reviews and Meta-Analyses Extension for Scoping Reviews) guidelines. A 3-step search strategy will be applied across multiple databases and gray literature sources. Eligible studies will include adults (aged ≥18 years) with a cancer diagnosis who are scheduled for elective treatment (surgery, radiotherapy, chemotherapy, immunotherapy, or hormone therapy). Interventions must involve eHealth or mHealth approaches supporting unimodal or multimodal prehabilitation activities such as exercise, nutrition, psychological support, or lifestyle modification. Outcomes of interest include functional fitness, quality of life, psychological well-being, treatment preparedness, recovery, adherence, and feasibility. Two independent reviewers will conduct title, abstract, and full-text screening, with disagreements resolved through discussion or consultation with a third reviewer. Data will be charted and presented in tables and figures and as a narrative synthesis. Critical appraisal using JBI tools will contextualize methodological quality but not exclude studies. Risk of bias will be assessed using the Cochrane Risk of Bias Tool for Randomized Trials version 2 (RoB 2) and Risk of Bias in Non-Randomized Studies of Interventions version 2 (ROBINS-I V2) tool. This will not be used to exclude studies, but to determine the quality of articles included.

**Results:**

The search strategy has been pilot tested and finalized. Database searches are scheduled to commence in March 2026, with study selection and screening anticipated to be completed by April 2026. Data analysis and synthesis are expected to begin in May 2026, and final results will be available by October 2026.

**Conclusions:**

This protocol outlines a rigorous and transparent approach to mapping the current evidence on technology-based prehabilitation in cancer care. By systematically characterizing intervention features, outcome domains, and evidence gaps, the review will provide an up-to-date evidence map to guide future research priorities, inform clinical implementation, and support the development of more standardized and inclusive prehabilitation pathways.

## Introduction

### Background

In the United Kingdom, the most recent available data showed that there were 346,217 cases of cancer reported in 2022 [1]. This figure was projected to grow to 420,000 by the end of 2025 [2]. Depending on the diagnosis, people affected by cancer are offered various treatments to manage or eradicate their cancer [3]. These treatments may include surgery, chemotherapy, radiotherapy, and immunotherapy, among others [3]. A population-based study analyzing the trends of cancer survival in England and Wales (1971‐2018) reported that the 10-year survival index of a person diagnosed with cancer in 2018 was 49.8% [4]. The cancer survival index has substantially improved since 1971 and is expected to continue improving [4]. This means that more people diagnosed with and living with cancer are expected to live longer. However, despite advancements in cancer treatment, patients still experience short- and long-term side effects that can significantly impact their quality of life (QoL) [5,6]. Cancer survivors experience significant long-term and late side effects and have unmet needs [6]. To support these patients, it is recommended that there be an increased awareness of these issues, to enable health care providers to improve personal care plans [5,6]. A qualitative study of 768 patients with varying types of cancer showed that after treatment, most patients’ QoL was influenced by their symptoms, and 82.3% of patients reported low QoL scores [7]. Additionally, a systematic literature review investigating length of life versus QoL considerations in patients with cancer showed that patients preferred to have a high QoL over a long life with low QoL [8]. This shows that the QoL of patients with cancer must be prioritized [8]. These studies suggest that an appropriate intervention needs to be investigated to improve the QoL of patients.

Prehabilitation is a comprehensive program that takes place before scheduled treatment and involves encouraging patients to complete healthy living and mindfulness interventions to help prepare them for treatment and improve their posttreatment recovery [9]. Prehabilitation can be unimodal or multimodal and typically involves patients completing physical exercise, nutritional, and psychological interventions [10]. Currently, prehabilitation is not recognized as standard care for National Health Service (NHS) patients with cancer; however, evidence shows that it can improve patients’ pretreatment functional capacity and preparedness, posttreatment recovery, and QoL [11-13]. The literature shows mixed outcomes for prehabilitation for presurgical patients with cancer when considering surgical complications and mortality; however, evidence still suggests that it is protective against surgery-induced decline [10,14]. Despite evidence outlining the benefits of prehabilitation, there are still challenges and inequalities impacting its success [15]. A narrative review reported that there are various socioeconomic, health literacy, adherence, and cultural challenges and inequalities impacting patients’ ability to complete prehabilitation programs [15]. There are many patients affected by these challenges and inequalities, meaning that they are less likely to engage in prehabilitation programs, despite often having worse perioperative outcomes [15]. This highlights that more research is needed to determine the most appropriate method of prehabilitation, and it must be implemented carefully to avoid widening existing inequalities [15].

The use of prehabilitation for patients with cancer receiving radical treatment has been widely accepted with proven benefits; however, patients requiring palliative care are often not considered for prehabilitation [16]. This is because palliative care is often viewed as end-of-life care [16]. A national survey investigating the use of prehabilitation for patients with esophagogastric cancer reported that both radical and palliative patients with cancer should be offered prehabilitation to manage cancer treatment preparation and recovery [17]. Fenemore et al [16] conducted a 12 to 24-week prehabilitation and rehabilitation program for patients with stage 4 lung cancer, as this patient group often experiences poorer outcomes and rapid health declines. They reported that the prehabilitation program had significant psychological benefits and found that prehabilitation is a feasible and acceptable program that significantly improves functional status and QoL [16]. This proves that future prehabilitation programs need to be inclusive and personalized to ensure all patients have equal opportunity to improve their psychological and health outcomes.

Traditionally, prehabilitation programs have been delivered by health care professionals in hospital-based settings, and recent studies have started to investigate the effectiveness of patients completing prehabilitation independently at home [11]. This is because traditional hospital-supervised prehabilitation is not accessible to all patients because of socioeconomic status, employment status, personal or family priorities, or geographic location [11]. However, recent reviews show that current research is unable to determine its true effect because lack of professional support, intervention, and monitoring is impacting patient adherence and compliance rates [13]. To reduce this issue, the use of technology to facilitate patients’ prehabilitation programs has been recommended [13].

Research shows that approximately 30% of the UK population own and use smartwatches and health apps daily for various health reasons, and this number is expected to rise [18]. Young adults (aged 18-34 years) are more likely to use wearables, whereas older adults (aged >55 years) are less likely to use them [18]. Mobile phone penetration in the United Kingdom is more than 85% [19]. Consequently, it is unlikely that smartphone-delivered prehabilitation will be a source of significant bias [19]. Technology has infiltrated people’s daily lives, and evidence shows that the use of technology was accelerated through the COVID-19 pandemic [20]. Research suggests that technology would improve adherence and compliance rates of prehabilitation programs and reduce barriers for patients restricted by socioeconomic, time, and geographical constraints [21,22].

A national survey of the provision of prehabilitation for patients with esophagogastric cancer found that 25% of respondents thought that prehabilitation should be delivered virtually in home-based settings [17]. However, there are concerns regarding the robustness of the current research designs and integration of technology [22,23]. Limited technology-based prehabilitation studies have been conducted globally, with mixed quality of evidence and risk of bias reported [22,23]. For clarity, we define “technology-based prehabilitation” as any prehabilitation intervention delivered wholly or partly using digital health technologies. Due to scant literature, risk of bias, and heterogenous reporting, a rigorous review is necessary before implementing or investigating technology-based prehabilitation to determine appropriate intervention design, ensure robustness and effectiveness, and meet patients’ needs. The purpose of the proposed scoping review is to identify and analyze the current technology-based prehabilitation interventions evidence to determine any gaps, highlight the most appropriate interventions and implementation designs, identify confounding factors that may impact prehabilitation, and examine the effectiveness and feasibility based on patient outcomes and feedback. This protocol will be the first step to achieving that.

A preliminary database search conducted in August 2025 of the Cochrane Database of Systematic Reviews, the Open Science Framework (OSF), JBI Evidence Synthesis, MEDLINE, Scopus, and Google Scholar found a limited number of published reviews and one registered review. These reviews focused on the accessibility and feasibility of technology-based prehabilitation for patients with cancer; they did not consider specific cancer sites or the effectiveness of specific prehabilitation interventions and placed a great emphasis on presurgery prehabilitation, with no mention of technology-based prehabilitation for patients before other cancer treatments such as radiotherapy or chemotherapy. Currently, there is limited high-quality evidence regarding technology-based prehabilitation for patients with cancer. Therefore, the purpose of this protocol is to register our intention to identify and analyze the current technology-based prehabilitation interventions evidence to determine gaps, highlight the most appropriate interventions and implementation designs, identify confounding factors that may impact prehabilitation, and examine the effectiveness and feasibility based on patient outcomes and feedback.

### Aims and Objectives

#### Aim

The aim of the proposed scoping review is to systematically map and synthesize the evidence on technology-based prehabilitation interventions for people with cancer, to identify current gaps in the evidence, highlight the most appropriate intervention designs, and determine the feasibility, acceptability, and accessibility based on patient outcomes.

#### Objectives

The objectives of this scoping review are as follows:

- Identify the existing eHealth and mobile health (mHealth) approaches to prehabilitation in cancer care, including wearables, mobile apps, telehealth, and online platforms
- Identify intervention characteristics such as the patient population, timing in the cancer treatment pathway, and the interventions delivered (eg, exercise, nutrition, psychological support, and lifestyle modification)
- Examine feasibility and acceptability outcomes, including recruitment, retention, adherence, patient engagement, usability, and satisfaction
- Assess reported patient outcomes such as physical function, psychological well-being, QoL, surgical readiness, morbidity, mortality, and recovery
- Explore equity and accessibility issues, including the influence of socioeconomic status, digital literacy, and demographic factors on participation and outcomes
- Identify knowledge gaps and future research priorities to inform the design of feasibility studies and support the integration of digital prehabilitation into routine cancer care

### Review Questions

#### Primary Question

This review addresses the following primary research question:

- What technology-based approaches have been used to deliver prehabilitation interventions for people living with cancer?

#### Secondary Questions

The secondary research questions addressed in this review are listed as follows:

- Which components of prehabilitation (eg, physical activity, nutrition, psychological support, and lifestyle modification) are delivered through these technologies?
- What evidence exists regarding the feasibility, acceptability, adherence, and accessibility of technology-based prehabilitation for people with cancer?
- What are the primary and secondary outcome variables?
- To what extent are equity-related factors (eg, socioeconomic status, digital literacy, and demographics) reported in studies of technology-based prehabilitation, and how do they influence outcomes?
- What are the key gaps in the evidence base, and what priorities should guide future research and intervention development?

## Methods

### Inclusion and Exclusion Criteria

The scoping review will be conducted following Joanna Briggs Institute (JBI) guidelines for scoping reviews in combination with the PRISMA-ScR (Preferred Reporting Items for Systematic Reviews and Meta-Analyses Extension for Scoping Reviews) checklist [24,25].

The inclusion and exclusion criteria were developed using the JBI guidance for scoping reviews population, concept, and context framework. Table 1 outlines the inclusion and exclusion criteria for this scoping review.

**Table 1.** Inclusion and exclusion criteria.

	Inclusion	Exclusion
Population	Adults aged 18 years or older with a confirmed cancer diagnosis preparing to undergo an elective cancer treatment (eg, surgery, chemotherapy, radiotherapy, hormone therapy, and immunotherapy)	Animal studies Studies involving participants younger than 18 years Populations without cancer
Concept	Technology-based prehabilitation interventions delivered prior to treatment, including any eHealth or mobile health approach (eg, apps, wearables, telehealth platforms, remote monitoring systems, and SMS text messaging–based interventions) used to support one or more prehabilitation components, such as exercise, nutrition, psychological support, or lifestyle modification	Rehabilitation instead of prehabilitation intervention
Context	All clinical and nonclinical settings, including hospital-supervised, community-based, and fully remote home-based interventions No restrictions will be applied regarding health care system, country, or delivery environment to map the full breadth of existing approaches	Review papers Conference papers Protocol papers

This review will include both unimodal and multimodal technology-based prehabilitation interventions. Unimodal interventions may focus on a single component such as exercise, nutrition, or psychological support, while multimodal interventions may combine 2 or more of these elements. Including both approaches will allow for comprehensive mapping of the evidence, enable comparison of intervention types, and support the identification of research gaps regarding feasibility, acceptability, accessibility, and patient outcomes.

Due to the proposed work being a scoping review, the outcomes of interest will be broad and exploratory, aiming to identify and map the range of outcome domains used to evaluate technology-based prehabilitation for patients with cancer scheduled to receive cancer treatment. All relevant domains reported across included study articles will be extracted, categorized, and synthesized to inform future research and intervention development. These outcome domains may include the following:

- Clinical outcomes (eg, postoperative complications, length of hospital stay, readmission rates, and physical function)
- Behavioral outcomes (eg, adherence, engagement, and activity levels)
- Psychological outcomes (eg, QoL, anxiety, self-efficacy, and patient confidence)
- Feasibility and implementation outcomes (eg, acceptability, usability, recruitment and retention rates, and barriers and facilitators)
- Service-level outcomes (eg, cost-effectiveness, workflow integration, and staff perspectives)

### Types of Sources

This scoping review will consider both experimental and quasi-experimental study designs, including randomized controlled trials (RCTs), non-RCTs, before and after studies, and interrupted time-series studies. In addition, analytic observational studies, including prospective and retrospective cohort studies, case-control studies, and analytic cross-sectional studies, will be considered for inclusion. This review will also consider descriptive observational study designs, including case series, individual case reports, and descriptive cross-sectional studies for inclusion.

### Scoping Review Methodology

The proposed scoping review will be conducted in accordance with the JBI methodology for scoping reviews [24]. The proposed scoping review has been registered with the OSF Registries as Technology-Based Prehabilitation for Cancer Patients Before Elective Treatment: A Scoping Review. It can be accessed at the OSF [26]. Any deviations from this protocol during the review will be documented with justifications and timestamps to ensure full transparency.

### Search Strategy

The search strategy will aim to locate both published and unpublished studies. A 3-step search strategy will be used in this review. First, an initial limited search of MEDLINE (PubMed) and CINAHL (EBSCO) database was undertaken to identify articles on the topic. The text words contained in the titles and abstracts of relevant articles and the index terms used to describe the articles were used to develop a full search strategy. This initial search strategy was conducted in September 2025. The search strategy was then further developed, adapted, and then applied for each database, including MEDLINE (EBSCO), CINAHL (EBSCO), PsycInfo (EBSCO), Scopus (Elsevier), Cochrane Central Register of Controlled Trials (Wiley), Web of Science (Clarivate), and AMED (EBSCO). An example search strategy and Boolean string used for CINAHL database are available in Multimedia Appendix 1. The search terms and Boolean string used in each database will be tailored to match platform-specific search requirements. Sources of unpublished studies, gray literature, and protocol registries to be searched include ProQuest Dissertations & Theses Global, OpenGrey, TRIP Database, and Overton.io databases, as well as ClinicalTrials.gov, and PROSPERO registries. The search strategy, including all identified keywords and index terms, will be adapted for each included database and/or information source. The reference lists of all included sources of evidence will be screened for additional studies.

During the search strategy design stage, GW received support from the Lancaster University Library team. They helped the researcher define their search strategy, identify MeSH (Medical Subject Headings) and Boolean terms, and select appropriate search databases. During the design stage it was decided to exclude Embase (Ovid) and Academic Search Ultimate (EBSCO) databases. Embase (Ovid) was excluded due to the substantial overlap with MEDLINE database, which is already included in the search strategy. Academic Search Ultimate was excluded because it is a broad multidisciplinary database with low precision for health-related topics, offering little unique yield beyond subject-specific sources. To balance comprehensiveness with feasibility, the review prioritizes specialist health databases most likely to capture relevant evidence. It was decided to exclude the gray literature databases Policy Commons, ERIC, conference proceedings, and white papers due to overlap with already included databases, limited relevance, and because they do not support reliable extraction of intervention design, outcomes, or implementation factors.

Only studies published since January 1995 will be included. This is because evidence suggests that global use of the internet for educational, health, military, and recreational use began in 1995 [27]. Older digital interventions (eg, pagers) will be included only to support historical mapping of conceptual evolution and not treated as directly equivalent to modern app- or wearable-based systems. Database and gray literature searches will be conducted in April 2026; specific dates of each search will be included in the final scoping review.

### Study Selection

Following the search, all identified citations will be collated and uploaded into EndNote (version 21; Clarivate Analytics). The citations will then be uploaded to Rayyan (Rayyan International Limited), an online platform designed for systematic review management, review automation, and data management. Rayyan will be used to remove duplicates. Following a pilot test, titles and abstracts will then be screened against the inclusion and exclusion criteria for the review. Authors will follow a unique set of screening instructions (Multimedia Appendix 2) to ensure that all articles are screened accurately. Reasons for exclusion of sources of evidence at full text not meeting the inclusion criteria will be recorded and reported in the scoping review. After the citations have been fully reviewed in Rayyan, they will be exported back into EndNote.

During the screening process, title, abstract, and full-text screening will be conducted by 2 reviewers (GW and LDH) to minimize selection bias. Both reviewers will pilot the eligibility criteria on a sample of records to ensure consistency in interpretation. Interrater reliability will be calculated and expressed via Cohen kappa statistic, with scores closer to 1 indicating stronger agreement. A value of>0.6 will be considered acceptable interrater reliability, as Landis and Koch identified this value as “substantial agreement” [28,29]. Any disagreements at either screening stage will be resolved through discussion; where consensus cannot be reached, a third reviewer (CJG) will adjudicate.

### Data Extraction

Data will be extracted from papers included in the scoping review using a custom data extraction tool (Multimedia Appendix 3). This tool has been piloted across 3 different data sources (a primary research article, a clinical trial protocol, and a feasibility research article). Data extracted will include specific details about the title, author, year, aims and purpose, participants, population and sample size, study design, intervention type (mHealth and eHealth), comparator, outcome measures, technological complexity, and other items from the Template for Intervention Description and Replication (TIDieR) checklist [30]. We distinguish 4 predefined technology complexities: low-complexity tools (eg, SMS reminders and static web resources), app-based interventions (mobile or tablet apps with structured content), wearable-enabled interventions (continuous or semicontinuous physiological or activity monitoring), and telehealth-mediated interventions (real-time remote supervision or coaching). We will also chart intervention intensity, from minimal contact to supported self-management and clinician supervised. The data extraction tool will be modified and revised as necessary during the review process of extracting and reviewing papers. Any modifications made will be detailed in the full scoping review. If any data are missing or additional data are required, authors of included articles will be contacted. Authors will follow a specific set of data extraction instructions (Multimedia Appendix 4) to ensure data extraction is accurately completed.

Although the JBI methodology for scoping reviews states that critical appraisal is not required [24], it will be included to provide context for interpreting findings. The evidence base for technology-based prehabilitation in cancer is heterogeneous and includes feasibility studies, pilot trials, RCTs, non-RCTs, and qualitative research. Assessing methodological quality using the relevant JBI critical appraisal checklists will help highlight strengths and limitations, particularly around feasibility, acceptability, and equity considerations [24]. The checklists that will be used include the RCT checklist for all RCT studies, the cohort study checklist for all non-RCTs and single-arm feasibility or pilot studies, and the qualitative study checklist for all qualitative studies. Appraisal results will not be used to exclude studies but will inform the interpretation of evidence, quality of evidence, and identification of research gaps. We will perform a series of structured subgroup syntheses, including comparisons based on technology category, intervention intensity, unimodal vs multimodal prehabilitation, and treatment pathway.

To ensure that feasibility, acceptability, and engagement outcomes are interpreted in a theoretically informed manner, data extraction and synthesis will be guided by 2 complementary frameworks: the Consolidated Framework for Implementation Research (CFIR) and the technology acceptance model (TAM) [31,32]. CFIR will be used to identify contextual and implementation-related determinants across 5 domains, with a focus on intervention characteristics, inner and outer setting factors, characteristics of individuals, and implementation processes [31]. TAM will be applied to capture user acceptance constructs, including perceived usefulness, perceived ease of use, behavioral intention to use the technology, and actual use or engagement [32]. To ensure analytical depth and avoid an overly descriptive synthesis, the data extraction tool was intentionally streamlined to prioritize CFIR and TAM constructs as the primary analytic domains, with clinical, psychological, and behavioral outcomes captured as secondary contextual variables. This enables coherent, theory-informed interpretation without diluting the analysis across an excessively broad range of outcome categories.

The Cochrane Risk of Bias Tool for Randomized Trials version 2 (RoB 2) and Risk of Bias in Non-Randomized Studies–of Interventions version 2 (ROBINS-I V2) tool will be used to provide a structured assessment of methodological quality across included studies [33]. Although formal critical appraisal is not mandatory for scoping reviews, incorporating these tools will enhance transparency and allow contextual interpretation of findings. The RoB 2 tool will be applied to included RCTs, as it provides a robust, standardized framework for evaluating internal validity across key domains such as randomization, blinding, and outcome reporting [33]. The ROBINS-I V2 tool will be used for included nonrandomized and quasi-experimental studies, enabling comparable assessment of potential bias due to confounding, selection, and measurement [33]. These tools are widely recognized for their methodological rigor and compatibility with mixed method evidence mapping. Findings from these bias assessments will be interpreted with explicit consideration of study quality, sensitivity of conclusions to high risk of bias, and identification of evidence gaps where methodological limitations are prevalent. The results from these tools will not be used to exclude studies but to provide context for interpreting the evidence base. Where sufficient studies exist within outcome domains, patterns in findings will be examined in relation to risk of bias ratings to avoid overinterpretation of weaker evidence. This method of reporting is consistent with the exploratory purpose of a scoping review.

Data extraction will be conducted independently by the first author (GW). To reduce the risk of error, a 2-step process (initial extraction followed by verification of accuracy against the source) will be adopted. Any uncertainties or ambiguities will be documented transparently and, where appropriate, resolved through consultation with the wider review team. This pragmatic approach ensures methodological robustness while acknowledging resource constraints.

### Data Analysis and Presentation

Data will be extracted and charted to directly address the review objectives and questions. Results will be presented in both tabular and graphical formats, supported by a narrative summary. Multimedia Appendix 5 outlines the piloted data chart that will be used.

The narrative summary will accompany the tables and figures, providing detailed descriptions of the evidence base, highlighting similarities and differences between interventions, identifying gaps in equity and inclusivity, and discussing implications for future research and clinical practice. Critical appraisal results will be presented in a separate table to contextualize the strengths and limitations of included studies, but not to exclude them, and a narrative summary will accompany the table to describe the findings. In addition, where possible, subgroup patterns will be explored (eg, unimodal vs multimodal interventions, cancer type, and prehabilitation before surgery vs other treatments).

Tabular presentation will be used when structured comparisons of study characteristics are required (eg, author, year, country, study design, cancer type, intervention type, timing in the cancer pathway, and outcomes measured). One large summary table will be used to present the key study characteristics and intervention details of all included studies. A separate table will be used to group reported outcomes into predefined domains (eg, physical, psychological, feasibility, patient experience, posttreatment outcomes, and clinical and service-level outcomes). The outcomes reported from the Critical Appraisal Skills Programme tools, the RoB 2, and ROBINS-I V2 tools will be reported separately in tabular format to ease visual interpretation.

Graphical or diagrammatic presentation (eg, bubble plots) will be used when visualization improves interpretation of distribution patterns. This method of presentation will be used to highlight the distribution of studies across cancer populations, treatment pathways, and countries, the types of technology-based prehabilitation interventions used, and the outcomes reported across the studies.

### Ethical Considerations

Due to the nature of this scoping review, ethical approval is not required.

### Inclusivity

The review author acknowledges the importance of equity, diversity, and inclusion in health research. The topic of this review-technology-based prehabilitation for people with cancer—has direct implications for equity, as access to digital health interventions can be influenced by socioeconomic status, digital literacy, language, geographic location, age, and other demographic factors.The review authors have a range of professional and research experience regarding oncology, prehabilitation, digital health, and patient-centered care. The review authors are committed to ensuring that the review considers the perspectives of diverse populations, including underserved and marginalized groups who may face barriers to accessing digital prehabilitation.Where possible, the authors will extract and report on equity-related data (eg, differences in intervention uptake or outcomes by age, sex, ethnicity, socioeconomic status, or digital literacy) to highlight gaps in inclusivity and inform future research.

## Results

**Figure 1.**
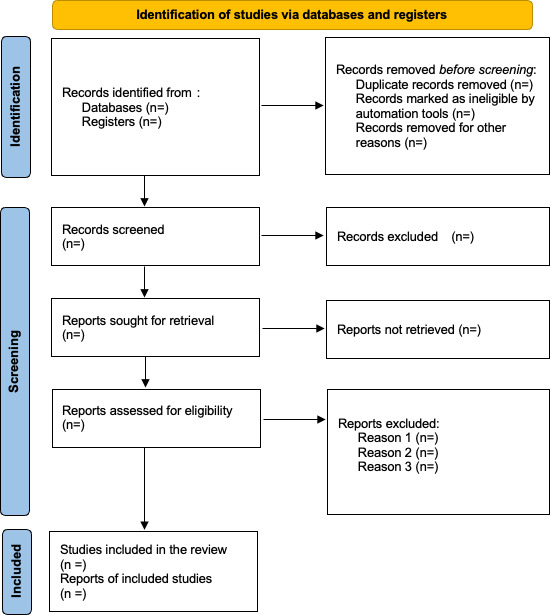
PRISMA (Preferred Reporting Items for Systematic Reviews and Meta-Analyses) 2020 flow diagram [25].

The protocol has been developed, and the search strategy has been pilot tested and finalized. Database searches are scheduled to commence in April 2026, followed by study selection and screening anticipated to be completed by May 2026. Data analysis and synthesis are expected to begin June 2026, and the results will be ready by November 2026. Results will be submitted for publication in November 2026. The results of the search and the study inclusion process will be reported in full in the final scoping review and presented using the PRISMA (Preferred Reporting Items for Systematic Reviews and Meta-Analyses) 2020 flow diagram (Figure 1; [25]). Throughout this period, database searches will be regularly updated to capture any newly published studies meeting the inclusion criteria. This project received no funding.

## Discussion

This scoping review, guided by this protocol, will map and critically contextualize a rapidly expanding yet methodologically diverse evidence base on technology-based prehabilitation in cancer care. Although several recent studies have explored aspects of digital prehabilitation, the field remains fragmented, with most existing interventions concentrated in presurgical pathways and centered on multimodal programs that primarily report feasibility and short-term physical or psychological outcomes. Current literature offers limited insight into digital approaches for patients preparing for radiotherapy, chemotherapy, immunotherapy, or hormone therapy and rarely examines implementation determinants, user acceptance, or digital equity considerations [34]. By synthesizing the full spectrum of technologies and treatment contexts and analyzing patterns using CFIR and TAM constructs, this review aims to provide a more nuanced and theoretically grounded understanding of where the evidence is converging, where substantial gaps remain—particularly in long-term outcomes, equity, and service-level implementation—and how these gaps can guide future research and inform the development of scalable, patient-centered digital prehabilitation pathways.

This protocol presents the methodology for a scoping review that will map and synthesize current evidence on technology-based prehabilitation interventions for patients with cancer. The review will highlight priorities for future research, which may include larger trials, longer-term and equity-focused evaluations, service-level outcomes, and greater inclusion of radiotherapy populations. These findings may inform standardized outcome reporting and clarify the potential role of digital health technologies in supporting prehabilitation and optimizing patient recovery.

This review protocol has several strengths, including a rigorous methodology following PRISMA-P (Preferred Reporting Items for Systematic Review and Meta-Analysis Protocols) guidelines and preregistration, clear eligibility criteria using a comprehensive search strategy of multiple databases, robust screening and data extraction with dual independent reviewers with calibration and Cohen κ for reliability, and the use of structured extraction forms and RoB 2 and ROBINS-I V2 for risk of bias assessment. The main limitations expected are those of the original studies. These include potential ambiguity in reporting, heterogeneity in study design and reporting, selective reporting, and lack of script or code transparency due to proprietary software. Results from this scoping review could identify areas for future research, such as lack of diversity in participants, potential for integration with telehealth and electronic health records, and device-specific or pathway-specific optimization.

Several limitations are anticipated. As a scoping review, a meta-analysis will not be conducted; therefore, findings will describe rather than quantify efficacy. Additionally, the heterogeneity of interventions, outcome measures, and patient populations may limit direct comparison across studies. Given the rapid pace of digital innovation, newly emerging technologies may also not be captured at the time of publication. Despite these limitations, this review will provide a comprehensive overview of existing research, identify evidence gaps, and guide future studies. It will contribute to understanding how digital approaches can enhance prehabilitation accessibility, adherence, and patient-centered outcomes within cancer care.

Findings will be disseminated through peer-reviewed publication, and conference presentations. They will also be shared through engagement with clinical, research, and patient stakeholder groups to support translation into practice and future research. Conclusions will emphasize findings from current digital health technologies.

## Supplementary material

10.2196/86610 Multimedia Appendix 1 Search strategy.

10.2196/86610 Multimedia Appendix 2 Screening instructions.

10.2196/86610 Multimedia Appendix 3 Data extraction tool.

10.2196/86610 Multimedia Appendix 4 Data extraction instructions.

10.2196/86610 Multimedia Appendix 5 Piloted data chart.

10.2196/86610 Checklist 1 PRISMA-P 2015 checklist.

## Abbreviations

CFIR: Consolidated Framework for Implementation Research
JBI: Joanna Briggs Institute
MeSH: Medical Subject Headings
mHealth: mobile health
NHS: National Health Service
OSF: Open Science Framework
PRISMA: Preferred Reporting Items for Systematic Reviews and Meta-Analyses
PRISMA-P: Preferred Reporting Items for Systematic Review and Meta-Analysis Protocols
PRISMA-ScR: Preferred Reporting Items for Systematic Reviews and Meta-Analyses Extension for Scoping Reviews
QoL: quality of life
RCT: randomized controlled trial
RoB 2: Risk of Bias tool for randomized trials version 2
ROBINS-I V2: Risk of Bias in Non-Randomized Studies–of Interventions version 2
TAM: technology acceptance model
TIDieR: Template for Intervention Description and Replication
