# Supplementary material for: Technology-Based Prehabilitation for Patients With Cancer Before Elective Treatment: Protocol for a Scoping Review
Source: JMIR Res Protoc. 2026 May 12;15:e86610. doi: 10.2196/86610 (PMC13167062; doi:10.2196/86610)
Supplement: Multimedia Appendix 1 [file resprot-v15-e86610-s001.docx]

### Appendix I: search strategy

|  | Database | CINAHL | |
| --- | --- | --- | --- |
|  | Date of search | 13^th^ October 2025 | |
| Search no. | Search terms | | Article results |
| S1 | (MH "Neoplasms") OR (MH "Neoplasms by Histologic Type") OR (MH "Neoplasms by Site") OR (MH "Neoplasms, Multiple Primary") | | 98,386 |
| S2 | ( (MH "Neoplasms") OR (MH "Neoplasms by Histologic Type") OR (MH "Neoplasms by Site") OR (MH "Neoplasms, Multiple Primary") ) OR TI ( cancer* OR oncolog* OR “patients with cancer” OR “oncology patients” OR “cancer patients” OR malignant* OR tumo* OR carcinoma OR “oncolog* disease” OR “oncolog* condition” OR “oncology-related illness” OR “living with cancer” OR “cancer condition” OR “cancerous tumo*” OR “neoplastic disease” OR “diagnosed with cancer” OR “person affected by cancer” OR “cancer care recipient” OR “oncolog* care patient” OR “undergoing cancer treatment” OR “solid tumour disease” ) OR AB ( cancer* OR oncolog* OR “patients with cancer” OR “oncology patients” OR “cancer patients” OR malignant* OR tumo* OR carcinoma OR “oncolog* disease” OR “oncolog* condition” OR “oncology-related illness” OR “living with cancer” OR “cancer condition” OR “cancerous tumo*” OR “neoplastic disease” OR “diagnosed with cancer” OR “person affected by cancer” OR “cancer care recipient” OR “oncolog* care patient” OR “undergoing cancer treatment” OR “solid tumour disease” ) | | 788,824 |
| S3 | (MH “Preoperative Care”) OR (MH “Prehabilitation”) | | 20,372 |
| S4 | ( (MH “Preoperative Care”) OR (MH “Prehabilitation”) ) OR TI ( prehab OR prehabilitation OR “pre-operative rehabilitation” OR “peri-operative rehabilitation” OR “before surgery rehabilitation” OR “before treatment rehabilitation” OR "pre-rehabilitation" OR “pre-treatment condition*” OR “presurgical optimi*” OR “functional prehab*” OR “oncol* prehab*” OR “pre-treatment care” OR “pre-surgery train*” OR “pre-surgery exercise train*” OR “pre-treatment train*” OR “pre-treatment exercise” OR “preoperative fitness” OR “pre-treatment intervention*” OR “pre-surg* intervention*” OR “physical prepar* for surgery” OR “physical prepar* for treatment” OR “prepar* for treatment” OR “prepar* for surgery” OR “resistance prehab*” OR “resistance exercise” OR “aerobic prehab*” OR “aerobic exercise” OR “pre-surgery physical activity” OR “pre-surgery exercise” OR “physical conditioning program” OR “lifestyle readiness program” OR “integrated pre-surgery care” OR “integrated pre-treatment care” ) OR AB ( prehab OR prehabilitation OR “pre-operative rehabilitation” OR “peri-operative rehabilitation” OR “before surgery rehabilitation” OR “before treatment rehabilitation” OR "pre-rehabilitation" OR “pre-treatment condition*” OR “presurgical optimi*” OR “functional prehab*” OR “oncol* prehab*” OR “pre-treatment care” OR “pre-surgery train*” OR “pre-surgery exercise train*” OR “pre-treatment train*” OR “pre-treatment exercise” OR “preoperative fitness” OR “pre-treatment intervention*” OR “pre-surg* intervention*” OR “physical prepar* for surgery” OR “physical prepar* for treatment” OR “prepar* for treatment” OR “prepar* for surgery” OR “resistance prehab*” OR “resistance exercise” OR “aerobic prehab*” OR “aerobic exercise” OR “pre-surgery physical activity” OR “pre-surgery exercise” OR “physical conditioning program” OR “lifestyle readiness program” OR “integrated pre-surgery care” OR “integrated pre-treatment care” ) | | 30,326 |
| S5 | (MH ”Digital Health”) OR (MH “Telehealth”) OR (MH “Telemedicine”) | | 38,951 |
| S6 | ( (MH ”Digital Health”) OR (MH “Telehealth”) OR (MH “Telemedicine”) ) OR TI ( smartwatch* OR “wearable tech*” OR “wearable device*” OR smartphone OR app* OR “mobile phone” OR online OR virtual OR wearable* OR “fitness track*” OR “digital health tool*” OR “tech* innovations” OR “digital intervention*” OR “health tech*” OR "eHealth" OR "mHealth" OR “digital platforms” OR “interactive device” OR “smart health tools” OR “health monitoring wearables” OR “health track*” OR “smart wristwear” OR “digital wrist device*” OR “health tracking smartwatch*” OR “health tracking device” OR “fitness companion” OR “health companion” OR “health and fitness companion” OR “real-time track*” OR “exercise wearable” OR telerehab* ) OR AB ( smartwatch* OR “wearable tech*” OR “wearable device*” OR smartphone OR app* OR “mobile phone” OR online OR virtual OR wearable* OR “fitness track*” OR “digital health tool*” OR “tech* innovations” OR “digital intervention*” OR “health tech*” OR "eHealth" OR "mHealth" OR “digital platforms” OR “interactive device” OR “smart health tools” OR “health monitoring wearables” OR “health track*” OR “smart wristwear” OR “digital wrist device*” OR “health tracking smartwatch*” OR “health tracking device” OR “fitness companion” OR “health companion” OR “health and fitness companion” OR “real-time track*” OR “exercise wearable” OR teleprehab* ) | | 1,600,252 |
| S7 | S2 AND S4 AND S6 | | 1,324 |
| S8 | (MH "Diagnosis") OR (MH "Diagnostic Imaging") OR (MH "Diagnostic Tests, Routine") | | 50,117 |
| S9 | ( (MH "Diagnosis") OR (MH "Diagnostic Imaging") OR (MH "Diagnostic Tests, Routine") ) OR TI ( biopsy OR imaging OR Needle OR "image-guided biopsy" OR "ultrasound" ) OR AB ( biopsy OR imaging OR Needle OR "image-guided biopsy" OR "ultrasound" ) | | 379,780 |
| S10 | (MH “Malnutrition”) | | 14,545 |
| S11 | (MH “Malnutrition”) OR TI ( "weight loss" OR "weight reduc*" OR obesity OR overweight OR malnurish* ) OR AB ( "weight loss" OR "weight reduc*" OR obesity OR overweight OR malnurish* ) | | 160,804 |
| S12 | S9 AND S11 | | 5,181 |
| S13 | S7 NOT S12 | | 1,321 |
| S14 | S13 (published from January 1995) | | 1,307 |
