# Supplementary material for: Technology-Based Prehabilitation for Patients With Cancer Before Elective Treatment: Protocol for a Scoping Review
Source: JMIR Res Protoc. 2026 May 12;15:e86610. doi: 10.2196/86610 (PMC13167062; doi:10.2196/86610)
Supplement: Multimedia Appendix 2 [file resprot-v15-e86610-s002.docx]

### Appendix II: Screening instructions

| 1. **Title and abstract screening** | - Assess whether the record is potentially relevant to the review objectives and research questions. - Records that clearly do not meet the inclusion criteria will be excluded. - Any articles that the screener is uncertain of based on the inclusion criteria will be included for full-text screening. |
| --- | --- |
| 1. **Full-text screening** | - Full-text articles will be retrieved for all records deemed potentially relevant. - The screener will assess eligibility using the predefined inclusion and exclusion criteria. - Reasons for exclusion at this stage will be recorded. |
| 1. **Process for resolving disagreements** | - All screening will be conducted independently. - If the screener is uncertain of any articles, they will request support from their master dissertation supervisors. This will ensure any disagreements or uncertainties are resolved. |
| 1. **Documentation** | - Screening decisions will be documented in a screening log within Rayyan. - A PRISMA flow diagram will summarise the selection process. |
