# Supplementary material for: Technology-Based Prehabilitation for Patients With Cancer Before Elective Treatment: Protocol for a Scoping Review
Source: JMIR Res Protoc. 2026 May 12;15:e86610. doi: 10.2196/86610 (PMC13167062; doi:10.2196/86610)
Supplement: Multimedia Appendix 3 [file resprot-v15-e86610-s003.docx]

### Appendix III: Data extraction tool

| Headings | Description | |
| --- | --- | --- |
| Custom study extraction information | Tier 1 | |
|  | Author, year, county |  |
|  | Study design |  |
|  | Population and sample size |  |
|  | Treatment pathway |  |
|  | Digital intervention type |  |
|  | Technological complexity |  |
|  | Intervention intensity |  |
|  | Clinical mediation level |  |
|  | CFIR | Complexity, adaptability, resources, workflow, participant benefit |
|  | TAM | Perceived usefulness, perceived ease of use, behavioural intention, engagement |
|  | Tier 2 | |
|  | Clinical outcomes |  |
|  | Psychological outcomes |  |
|  | Behavioural outcomes |  |
|  | Tier 3 | |
|  | Equity/accessibility outcomes |  |
| TIDieR checklist | Why |  |
|  | What |  |
|  | Who provided |  |
|  | How |  |
|  | Where |  |
|  | When and how much |  |
|  | Tailoring |  |
|  | Modifications |  |
|  | How well |  |
