# Supplementary material for: Technology-Based Prehabilitation for Patients With Cancer Before Elective Treatment: Protocol for a Scoping Review
Source: JMIR Res Protoc. 2026 May 12;15:e86610. doi: 10.2196/86610 (PMC13167062; doi:10.2196/86610)
Supplement: Multimedia Appendix 4 [file resprot-v15-e86610-s004.docx]

### Appendix IV: Data extraction instructions

| 1. **Read** | Read the full text of each included study carefully before beginning extraction. |
| --- | --- |
| 1. **Complete the extraction tool** | Complete all parts on the extraction tool. |
| 1. **Record all parts of the data** | Record data verbatim from the study where possible; if interpretation is required, note this clearly. |
| 1. **Missing data** | Do not leave any parts of the data extraction tool blank. Use “Not reported” if data is unavailable. |
| 1. **Check consistency** | Check consistency with the inclusion/exclusion criteria and note any uncertainties that may need discussing. |
| 1. **Data saving** | Save the extracted data in a designated file, following version control procedures. |
