# Supplementary material for: Technology-Based Prehabilitation for Patients With Cancer Before Elective Treatment: Protocol for a Scoping Review
Source: JMIR Res Protoc. 2026 May 12;15:e86610. doi: 10.2196/86610 (PMC13167062; doi:10.2196/86610)
Supplement: Multimedia Appendix 5 [file resprot-v15-e86610-s005.docx]

### Appendix V: Piloted data chart

| Author, year, country | Study design | Population | Intervention type | Intervention components | Timing in cancer pathway | Comparison | Feasibility and acceptability outcomes | Patient outcomes | Equity/Accessibility factors | Key findings |
| --- | --- | --- | --- | --- | --- | --- | --- | --- | --- | --- |
|  |  |  |  |  |  |  |  |  |  |  |
